# Identification and determination of myriocin in *Isaria cicadae* and its allies by LTQ-Orbitrap-HRMS

**DOI:** 10.1080/21501203.2017.1383319

**Published:** 2017-10-03

**Authors:** Wen-ming Cheng, Qun-lin Zhang, Ze-hua Wu, Zhi-yong Zhang, Yi-ru Miao, Fan Peng, Chun-ru Li

**Affiliations:** a School of Pharmacy, Anhui Provincial Key Laboratory of Bioactivity of Natural Product, Anhui Medical University, Hefei, China; b Zhejiang BioAsia Institute of Life Sciences, Pinghu, China; c Anhui Provincial Key Laboratory of Microbial Control, Anhui Agricultural University, Hefei, China

**Keywords:** *Isaria cicadae*, myriocin, determination, LC-MS, LTQ-Obitrap-HRMS

## Abstract

A hybrid linear ion trap-quadrupole-Orbitrap high-resolution mass spectrometry (LTQ-Orbitrap-HRMS) was used to qualitatively and quantitatively analyse the myriocin in *Isaria cicadae* and its allies. The samples were prepared with 95% methanol for 30 min by ultrasonic-assisted extraction. The target compound was purified by ODS solid-phase extraction (SPE) column. The enriched samples were identified by mass spectrometry. The results showed that the contents of myriocin in both wild and artificial *Isaria cicadae* were below the detection limit, while a strain of *Ophiocordyceps longissima* and *Cordyceps cicadae* Shing (Dujiaolong), both closely related to the *Isaria cicadae*, and its asexual mycelia are rich in myriocin. It suggests that it may be wrong to consider *C. cicadae* as *I. cicadae*’s teleomorph in Genbank or Mycobank in many published reports based on chemical classification, and the species rich in myriocin is probably not *Isaria cicadae*.

## 1. Introduction

Vegetable cicada, a traditional Chinese herbal medicine, is a Cordycipitoid fungus named *Isaria cicadae* (≡*Paecilomyces cicadae* (Miquel) Samson) parasitising cicada nymphs. Vegetable cicada is a worldwide species, distributed in China, Southeast Asian countries, the United States and many other nations and regions. It is often confused with its allies, especially *Cordyceps cicadae*. But *I. cicadae* and *Cordyceps cicadae* are belong to different species. Their fruiting bodies or stroma are different in texture, colour and morphology. The stroma of *Cordyceps cicadae* occur from the head of the host, horn-like, dark brown or purple brown, fleshy, erect, solitary, unbranched. The synnemata or coremia of *I. cicadae* grow densely from the front of the host, especially the head, cinnamon, branched into cockscomb-like or broccoli-like head.

Vegetable cicada is an edible and medicinal fungus with high nutritional value and pharmacological function. It contains a variety of physiologically active substances, including nucleosides, cyclic peptides, polysaccharides, alcohols, sterols and organic acids (Tan et al. 2007; Zhang and Xuan 2007, 2008), which play an important role in health care, such as regulating immunity, improving renal function, modulating lipid metabolism, reducing blood pressure and blood sugar, possessing anti-tumour, anti-fatigue, analgesic and hypnotic activities (Chen 1991; Weng et al. 2002; Lu et al. 2006; Song et al. 2007; Zhu et al. 2011, 2016; Wang et al. 2014). Natural resources of vegetable cicada are become more and more popular for health care and too insufficient to meet the market demand. However, the quality of wild vegetable cicada is questioned because of quality and safety problems due to various origins, different harvest time, possibility of exceeding heavy metals and microorganic contaminant. The artificial cultivation of vegetable cicada has become a hot issue and drawn more and more attention. At present, artificial cultivation of vegetable cicada at large scale has been successful. So it is important to confirm the reliability of fungus origin and develop the method of assessing the quality of vegetable cicada products.

Many studies on the composition of vegetable cicada have been reported, but it is still controversy on whether it contains myriocin or not (Wang et al. 2009; Yu et al. 2009). Myriocin, a fungal metabolite, is a potent inhibitor of serine palmitoyltransferase (SPT), which is the key rate limiting enzyme in sphingolipid biosynthesis. Myriocin exhibits antifungal and strong immonosuppressive activity. However, long-term use of myriocin may produce adverse effects on health by modulating biological functions of cells as a result of persistent disruption of sphingolipid metabolism (He et al. 2004). Several analytical methods to detect myriocin, such as HPLC-light scattering detection (Wang et al. 2009), HPLC-fluorescence detection with a complex precolumn derivatisation by fluorenylmethyl-chloroformate (Yu et al. 2009), HPLC-OPA-UV (Yu et al. 2012) and HPLC-MS (Campisi et al., 2017), have been published. With the rapid development of high-performance liquid chromatography-mass spectrometry, a hybrid linear ion trap-quadrupole-Orbitrap high-resolution mass spectrometry (LTQ-Orbitrap-HRMS) combined with Fourier-transform technology, offers ultra-high resolving power with resolution up to 100,000. Moreover, the multiple precursor ions were fragmented and the precursor ions were detected with a mass tolerance of less than 5 ppm. It can realise the rapid identification and sensitive analysis of the chemical composition in traditional Chinese medicine, and provides a highly efficient and reliable method of identification and quantification of trace components. Therefore, in the present study, LTQ-Orbitrap-HRMS was used to qualitatively and quantitatively analyse the myriocin in vegetable cicada and its related *Cordyceps*.

## 2. Materials and methods

### 2.1. Materials and reagent

The materials of wild vegetable cicadas and *Cordyceps cicada* were obtained from the local market. The artificial fruiting bodies and substrate of vegetable cicada were produced by Zhejiang BioAsia Pharmaceutical Co., Ltd (Pinghu, China). The artificial fruiting bodies and mycelia of *Ophiocordyceps longissima* were provided by Research Center for Entomogenous Fungi of Anhui Agricultural University (Hefei, China). All materials were powdered on a FYl35 herbal mill (Tianjin Taisete Instrument Co., Ltd., China). All fungal materials were identified by Prof. Chun-ru Li and all voucher specimens were deposited at Anhui Provincial Key Laboratory of Bioactivity of Natural Product, Anhui Medical University.

Standard myriocin was purchased from Sigma (St. Louis, MO, USA). Acetonitrile and methanol were obtained from Merck (Darmstadt, Germany). Formic acid was produced by Tedia (Fairfield, OH, USA). All solvents above were of HPLC-MS reagent grade. Deionised water was prepared using a Millipore Milli-Q Plus system (Bedford, MA, USA).

### 2.2. Methods

#### 2.2.1. Preparation of standard and sample solutions

Standard myrion 1.0 mg was accurately weighed on a BP211D type 1/(10 million) electronic analytical balance (Sartorius, Germany), and transferred to a 10-mL volumetric flask and made up to volume with methanol. The solution was filtrated through a 0.22 μm membrane prior to analysis.

Quantitatively weighed 1.0 g of dried Cordyceps powder was sonicated with 95% methanol (3 × 10 mL) for 40 min at 40°C. The extract solutions were combined and concentrated on a rotary evaporator under reduced pressure at 50°C after filtration. The residue was dissolved in 2 ml 50% methanol. After centrifugation at 12 000 r/min for 10 min with a low-temperature high-speed centrifuge (Thermo-Fisher, USA), the supernatant was transferred onto the ODS-solid-phase extraction (SPE) column (500 mg/3 ml, 40–60 μm, HongPu Scientific, Ningbo, China), which was pretreated with 10 ml methanol and 5 ml water (Yu et al. 2009). The column was washed with 10% methanol (1 × 3 mL) and 50% methanol (2 × 3 mL) successively. After the last flow was stopped in the column for approximately 20 min, 80% methanol (2 × 3 mL) was used to elute myriocin. The eluent was combined and evaporated in a water bath at 50°C. The residue was dissolved in 1 ml methanol. After centrifugation at 12,000 r/min for 10 min, the supernatant was transferred to a 1-mL eppendorf tube. After filtration through a 0.22-μm membrane, The solutions were diluted 100 times and vortex-mixed on a XW-80A scroll mixer (Shanghai Huxi Analysis Equipment Factory, China).

#### 2.2.2. HPLC-LTQ-Orbitrap-HRMS analysis

HPLC-HRMS analysis was performed on an Ultimate 3000 HPLC system acquired from Thermo Fisher Scientific (San Jose, CA, USA) that consisted of an autosampler equipped with a column oven, a tray compartment cooler, and a high-pressure quaternary gradient pump with an on-line solvent degasser, all piloted by Xcalibur 2.1 software. The chromatographic separation was performed on a Thermo Hypersil BDS C18 (I.D. 2.1 mm × 150 mm, 2.4 μm), column temperature 40°C; mobile phase: water (A) – methanol (B). Gradient elution: 0~5 min, 50% B; 5.1 ~ 8.0 min, 50~100% B; 8.1~15 min, 100% B; 15.1~17 min, 100~50% B; 17.1~20 min, 50% B; flow rate at 0.2 mL/min; injection volume at 2 μL.

HPLC system was coupled with a LTQ Orbitrap XL high-Resolution mass spectrometer (Thermo Fisher Scientific, USA). For identification purposes, collision-induce dissociation (CID) was applied for MS experiments. The system was equipped with an electrospray ionisation (ESI) source that was operated in negative ion mode with the following conditions: source voltage at 4 kV; capillary temperature and voltage at 275.00°C and −35.00 V, respectively; tube lens voltage at −40 V; evaporator temperature at 300.00°C; cluster cracking voltage at 9 V; collision energy at 35 V; collision energy rolling range at 15 V. Nitrogen was used as the sheath gas and helium as auxiliary gas with a flow rate of 20.00 and 5.00 arbitrary units, respectively. Spectra were recorded in the range of m/z 100–1000 with a resolution of 30,000. Quantitative scanning mass was in the range of 300.00~600.00.

An external calibration of the equipment for mass accuracy was carried out according to the manufacturer’s guidelines the day before the analysis. The specific peak was identified by comparison with standard myriocin from the retention time, MS and MS/MS analysis. Product ions and their relative proportions were evaluated and compared with the standard. For quantification, full-scan MS mode was applied, and then extracted ion chromatograms of the ions of interest were generated from their theoretical exact masses within a mass tolerance of 5 ppm. After integration of the peaks, their respective areas were used for quantification. Finally, the quantification was performed using the external calibration method.

#### 2.2.3. Validation of the method

Standard stock solution was prepared and diluted to a series of appropriate concentrations for the construction of calibration curves. Every calibration curve covered at least six concentration levels. Each of the concentration levels were injected in triplicate and linear regression was constructed on plots of peak areas against the concentration of each analyte. In order to determining the LODs and LOQs, the diluted solutions of the analyte were further diluted with mobile phase to give a series of concentrations. The LOD and LOQ under the present conditions were defined as the concentration of target compound giving S/N = 3 and 10.

To examine the precision of the method, the intra-day precision was calculated by testing the known concentration of the analyte in six replicates during a single day and inter-day precision was tested through replicate analysis of standard on three consecutive days. Five different working solutions prepared from the same sample (*O. longissima*) were analysed to determine the repeatability. The per cent value of relative standard deviation was chosen as a measure of precision and repeatability.

The standard solution was added according to three levels (1:0.8, 1:1, 1:1.2) to a precisely weighed portion of *O. longissima* to test the reproducibility of the method. The mixtures were extracted and analysed under the same methods mentioned above. Recovery calculation formula is used for calculating the per cent value of recoveries: Recovery (%) = (amount tested – original amount)/amount spiked ×100%.

## 3. Results

**Figure 1.**
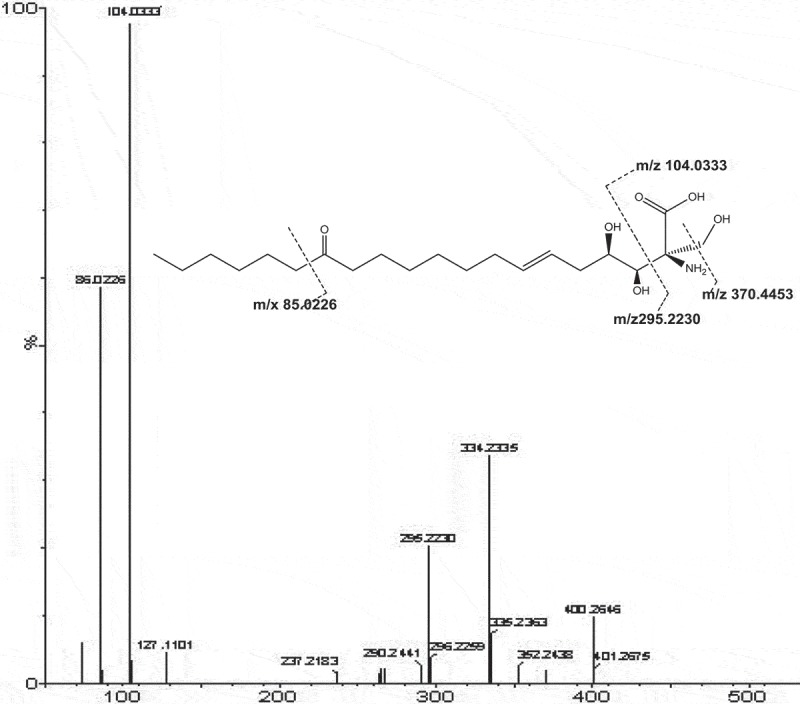
Mass spectrum of myriocin.

The samples were prepared according to the method under 2.2.1, and the chemical constituents of the vegetable cicada were qualitatively analysed by HPLC-LTQ-Orbitrap-HRMS according to the chromatographic conditions under 2.2.2. The (-) ESI-MS total ion flow diagram (TIC) are shown in Figure 1. It can be observed that LC-MS profiles of vegetable cicada extract showed good separation among the chromatographic peaks of the composition. By detecting the retention time and the precise molecular weight of the targeted peak, combined with the secondary mass spectrometry to crack the fragment information, using the standard comparison, through searching the SciFinder, ChemSpider etc., myriocin of Cordyceps samples could be quickly identified.

The external standard method was employed to calculate the contents of myriocin in the tested samples. The standard solutions were diluted with methanol to five different concentrations for the construction of calibration curves. The peak area (x) was plotted against the injection concentration (y, μg·mL^−1^). The linear equation was calculated as y = 1E-07 x –0.109 (R^2^ = 0.9992). The parameters of LOD and LOQ were 0.005 μg·mL^−1^ and 0.015 μg·mL^−1^, respectively.

The intra-day and inter-day precisions of the present method were calculated by analysing the standard solution under the experimental conditions. The RSD values of them were 2.52 and 2.62%, respectively, indicating good precision of the instrument. The RSD value of stability of the analyte in 24 h at room temperature (*n* = 6) was 2.85%, showing good stability of the test solution within 24 h. Furthermore, the sample solution was prepared in parallel (*n* = 6) to evaluate the repeatability. The average recovery rate was 96.15~98.64%, with the RSD values from 1.68 to 2.69%, indicating that the reproducibility of the experimental method was good.

**Figure 2.**
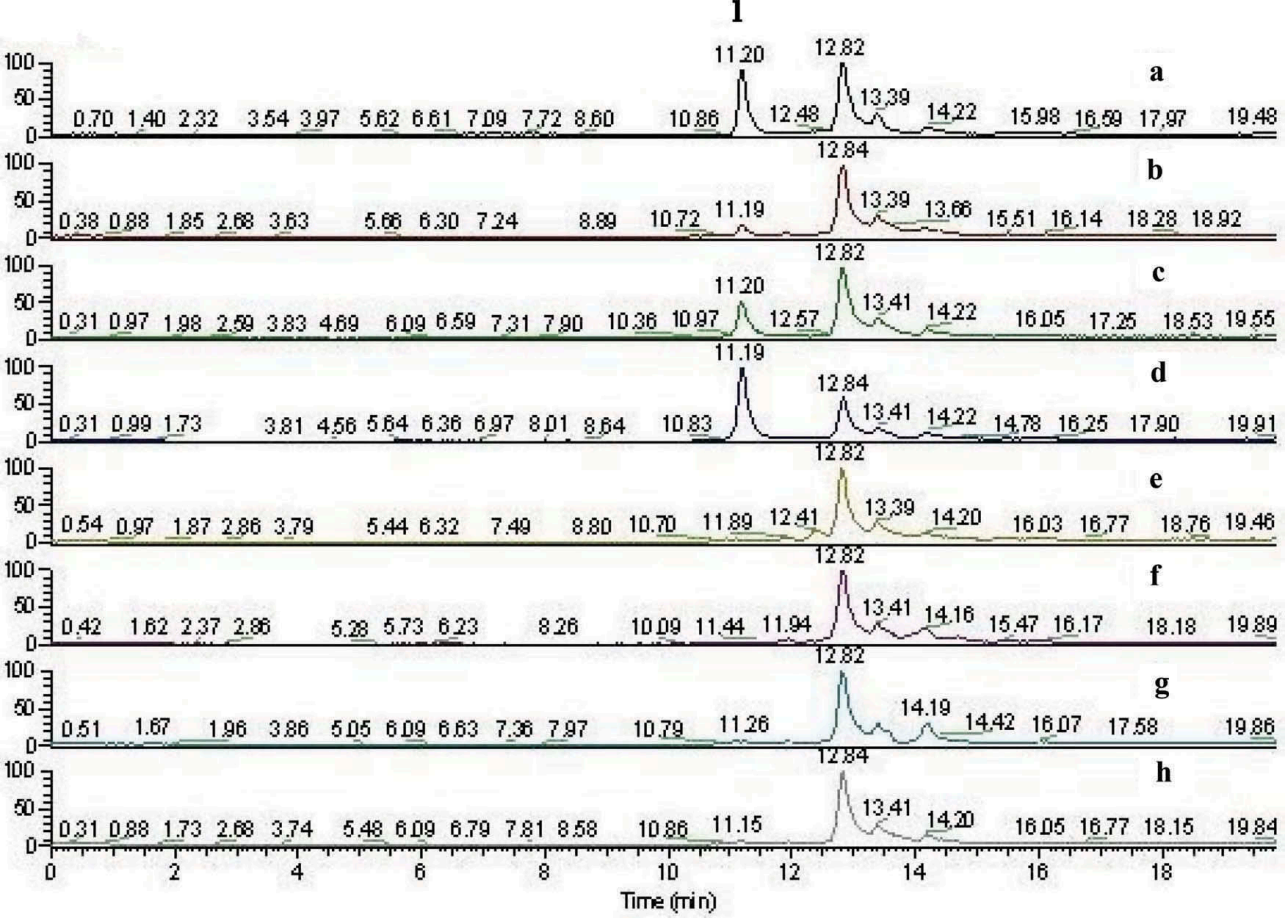
Ion map(SIM)of vegetable cicada and its allies. (a): Myriocin (1: 11.20 min); (b): *Cordyceps cicadae*; (c): Fruiting body of *Ophiocordyceps longissima*; (d): Mycelium of *O. longissima*; (e): Fruiting body of vegetable cicada; (f): Substrate of vegetable cicada; (g): Wild vegetable cicada (Anhui); (h): Wild vegetable cicada (Henan).

This validated HPLC-LTQ-Orbitrap-HRMS method was used for the quantification analysis of myriocin in 7 batches of Cordyceps under the SRM mode. The negative ion spectra shows intense signal at m/z 400.2646 for myriocin. The main fragment in the low m/z spectral region derive from the threonine portion (formation of deprotonated serine, m/z 104.0333, C_3_H_6_NO_3_^+^), and fission of the bonds close to the C-14 carbonyl group yields a minor fragment at m/z 85.0226, C_6_H_13_^+^). The first-generation loss of the elements of formaldehyde (CH_2_O; −30 Da) and subsequent loss of two water molecules (H_2_O, H_2_O; −18–18 Da) were yielded from the precursor ion [M-H]^−^ (m/z 400.2646) to derive the fragments at m/z 370.4453, m/z 352.2433 and m/z 334.2335, respectively, as shown in Figure 1. The content of myriocin was calculated by its calibration curve, and the quantification results were presented in Figure 2 and Table 1. By comparison of the retention time and fragmentation pattern with a reference standard, myriocin was identified and confirmed, for the target peak showed the same retention time at 11.20 and the same fragmentation as the standard (Figure 2). As presented in Table 1, myriocin was quantified in sample D, C and B at the levels of 189.36, 49.49, 9.80 μg/g, respectively. But it was very low and under the limit of determination in sample E, F, G and H, which were classified as *Isaria cicadae*.

**Table 1.** The content of myriocin in vegetable cicada and its allies.

Sample	Origin	Source	Content(μg/g)
B	Wild Cordyceps cicadae	Yunnan	9.80
C	Fruiting body of *O. longissima*	Anhui Agricultural University	49.49
D	Mycelium of *O. longissima*	Anhui Agricultural University	189.36
E	Fruiting body of vegetable cicada	Zhejiang Bioasia Institute of Life Sciences	UD
F	Substrate of vegetable cicada	Zhejiang Bioasia Institute of Life Sciences	UD
G	Wild vegetable cicada	Anhui	UD
H	Wild vegetable cicada	Henan	UD

UD: under the limit of determination.

## 4. Discussion and conclusion

Myriocin was initially extracted from the culture broth of *Isaria sinclairii* parasitised cicada niphas by Professor Fujita of the Japanese University of Tokyo in 1994 (Fujita et al. 1994). We previously reported that the ethyl acetate extract of *Cordyceps cicadae* fermented broth had a significant inhibitory effect on *Candida albicans*, which was selected as the best indicator of the antibacterial activity and the antifungal compound isolated from the fermentation broth of *C. cicadae* was myriocin (Hu et al. 2016). The myriocin in RCEF3666, RCEF3890 and RCEF3891, 3 strains of *Ophiocordyceps longissima*, and 1 strain of *Isaria cicadae* RCEF6212 and its mycelia was also identified, and the results showed that only RCEF3891 (*O. longissima*) contained myriocin (Zhang 2014). Then the artificial cultures of a large number of *Isaria cicadae* strains deposited at the laboratory were detected, and myriocin were still not detected. This result was consistent with the previous report (Wang et al. 2009).

Yu et al. (2009) have reported that myriocin was detected in the wild sample from Chongqing (Latin name they used *Cordyceps cicadae*, but Chinese name they called vegetable cicada) and in its cultures, but not in the wild samples from Guizhou and Sichuan. In view of the fact that the content of myriocin in dozens of vegetable cicada from different laboratories (including Yu’s laboratory) and their cultures is less than the detection limit, so it may not be simply attributed to the differences among strains. It may be concluded that the strain is most likely to be *Isaria sinclairii* but not *Isaria cicadae* (vegetable cicada), or one of *Cordyceps cicadae, Ophiocordyceps longissima* and its similar species *O. sobolifera. O. sobolifera* and *Cordyceps cicadae* (Dujiaolong) have been mistaken for teleomorph of *Isaria cicadae*, so that it is the possibility of confusing the three species. *Isaria cicadae* has its own teleomorphic state, not *O. sobolifera* or *Cordyceps cicadae* (Dujiaolong).

It is interesting to note that there is a significant difference containing myriocin among the strains of *O. longissima*, and even the content of myriocin in different cultures of the same strain is different, e.g. the content of myriocin in the stroma of RCEF3891 was 49.49 μg/mg, while the content of myriocin in its synnema was 189.36 μg/mg, maybe due to having too many conidia in the synnema.

The ability of different species of *Cordyceps* to be preserved in phylogeny is closely related to their antifungal activity (Li 1988). Also, myriocin content in some species or strains is low, easy to be lost during the separation process and more attention to a variety of combined methods should be paid.

In this study, HPLC-LTQ-Orbitrap-HRMS technique was used to analyse and confirm the myriocin in vegetable cicada and its allies. It suggests that the species which contain high content of myriocin may be not *Isaria cicadae* but its allies and the results provided the basis to study the pharmacological application of drug further.
